# Risk factors for refractory Kawasaki disease: clinical records of the paediatric clinic of palermo

**DOI:** 10.1186/1546-0096-12-S1-P359

**Published:** 2014-09-17

**Authors:** Maria Cristina Maggio, Eugenia Prinzi, Giovanni Corsello

**Affiliations:** 1 Pro.Sa.M.I. "G. D'Alessandro", University of Palermo, Palermo, Italy

## Introduction

Kawasaki disease (KD) is an acute, self-limited febrile illness that mainly affecting small- to medium-sized vessels and occurs in early childhood. The etiology is currently unknown, however it likely results from an immunologic response triggered by microbial agents, with documented genetic susceptibility. Intravenous administration of immunoglobulin (IVIG) is the gold standard therapy for coronary arteritis in the acute phase of KD; some patients do not respond to IVIG and coronary aneurysms continue to develop in 5%. The most serious complications are coronary vasculitis and aneurysms. 15% of these patients do not respond to IVIG (Refractory KD:RMK) and have a higher risk of aneurysms.

## Objectives

To predict RKD, Kobayashi et al. suggested a score system including: age, gender, days of the disease at IVIG start, neutrophyls and platelets count, AST, CRP, Na. Recent reports suggest the utility of a combined treatment with IVIG and steroids or Infliximab to reduce the risk of coronary involvement in RKD. We analyzed Kobayashi criteria and we also considered D-dimer and gamma-GT, to elevate the sensitivity of the score.

## Methods

We analyzed the clinical records of the Paediatric Clinic of Palermo, since January 2008 till april 2014: 65 patients with KD (68% Typical KD: TKD; 4% Atypical KD: AKD; 28% incomplete KD: IKD).

## Results

33 (51%) are males; 32 (49%) females, with a M:F ratio of 1.03, lower of that reported (1.5-1.7). Age at the diagnosis was 2.1±1.8 years. The fever at the admittance was since 5.39±2.40 days; the most frequently relieved symptoms were: conjunctivitis (88%), stomatitis (85%), rash (71%). IVIG were administered 6.7±2.5 days later the fever start. Defervescence occurred 36.8±24 hours after IGEV. 53 patients (82%) received 1 dose of IVIG; 18% had a RKD, with persistent fever after IVIG. 11% responded to the second dose; 5% to three doses; one patient to Infliximab. 15 patients (23%) had aneurisms (20% in responders; 36% in RKD). 64% (7/11) of RKD vs 44% (24/54) of responders showed cardiac involvement. Precocious pericarditis was associated with and more precocious than coronary involvement.

Age was lower in RWD (1.4±1.1). The days of fever pre-IVIG were inversely correlated with IVIG doses. 5/11(45%) of RKD received the first dose <5days. None had platelet count <30.000 mm^3^ or % neutrophils >80%. 2 had AST>100. 82% (9/11) of RKD had Na<133 vs 26% of responders. Leucocytes were significantly directly correlated with fibrinogen pre-IVIG (p:0.001). IVIG doses were directly correlated with CRP post-IVIG (p:0.044), gamma-GT pre-IGEV (p:0.008), D-dimer pre-IGEV (p:0.014). In RKD D-dimer was 1188±724 ng/ml, significatly higher than in respoders (798±662).

Days of fever at the recovery were inversely correlated with pre-IVIG AST (p:0.034), and ALT (p:0.013).

## Conclusion

Our data well correlate with Kobayashi score. However we stress the role of D-dimer and gamma-GT as prognosic criteria for RKD. The role of neutrophils, platelets reduction is decreased.

## Disclosure of interest

None declared

